# Building Social Support and Moral Healing on Nursing Units: Design and Implementation of a Culture Change Intervention

**DOI:** 10.3390/bs14090796

**Published:** 2024-09-10

**Authors:** Timothy J. Usset, Cassandra Godzik, J. Irene Harris, Rebecca M. Wurtz, Jeffrey M. Pyne, Stephanie W. Edmonds, April Prunty, Rebecca J. L. Brown, Shoshana H. Bardach, Joel M. Bradley, Christopher L. Hubble, Brant J. Oliver, Renee L. Pepin, Joseph Currier, Andrew J. Smith

**Affiliations:** 1 School of Public Health, University of Minnesota, Minneapolis, MN 55455, USA; 2 VA Maine Healthcare System, Augusta, ME 04330, USA; 3 Geisel School of Medicine at Dartmouth, Hanover, NH 03755, USA; 4 Dartmouth-Hitchcock Medical Center, Lebanon, NH 03766, USA; 5 Department of Psychology, University of Maine, Orono, ME 04469, USA; 6 Center for Mental Health Outcomes Research, Central Arkansas Veterans Healthcare System, North Little Rock, AR 72114, USA; 7 Psychiatric Research Institute, University of Arkansas for Medical Sciences, Little Rock, AR 72205, USA; 8 Abbott Northwestern Hospital, part of Allina Health, Minneapolis, MN 55407, USA; 9 VA Minneapolis Healthcare System, Minneapolis, MN 55455, USA; 10 The Dartmouth Institute for Health Policy & Clinical Practice, Hanover, NH 03755, USA; 11 Department of Psychology, University of South Alabama, Mobile, AL 36688, USA; 12 Lyda Hill Institute for Human Resilience, University of Colorado-Colorado Springs, Colorado Springs, CO 80918, USA

**Keywords:** nurses, social support, peer support, moral injury, moral distress, resilience, evidence-based quality improvement, organizational intervention

## Abstract

The healthcare industry continues to experience high rates of burnout, turnover, and staffing shortages that erode quality care. Interventions that are feasible, engaging, and impactful are needed to improve cultures of support and mitigate harm from exposure to morally injurious events. This quality improvement project encompassed the methodical building, implementation, and testing of RECONN (Reflection and Connection), an organizational intervention designed by an interdisciplinary team to mitigate the impact of moral injury and to increase social support among nurses. This quality improvement project was conducted in a medical intensive care unit (MICU) in a rural, academic medical center. We employed an Evidence-Based Quality Improvement (EBQI) approach to design and implement the RECONN intervention while assessing the feasibility, acceptability, and preliminary effectiveness via surveys (*n* = 17). RECONN was found acceptable and appropriate by 70% of nurses who responded to surveys. Preliminary effectiveness data showed small to moderate effect sizes for improving social support, moral injury, loneliness, and emotional recovery. Further evaluation is warranted to establish the effectiveness and generalizability of RECONN to other healthcare settings.

## 1. Introduction

Healthcare workers (HCWs) are in the midst of a post-pandemic reckoning, with burnout, turnover, and attrition taking a toll on their well-being [1]. Pre-existing mental and occupational health distress among HCWs (e.g. burnout), was experienced by 40–60% of clinicians prior to the pandemic, and recent literature [2,3,4,5] demonstrate distress has worsened since then. One cause of this distress—before, during, and since the pandemic—is moral injury, the psychological, behavioral, social, and spiritual aftermath of exposure to events that transgress deeply held beliefs [6,7,8,9]. First studied in military/veteran populations, moral injury is linked to depression, anxiety, anger, self-harm, and social problems related to the guilt, shame, existential crisis, and the loss of trust that occur when HCWs are unable to provide the care they know their patients need [8,10,11].

Recent studies indicate that 17–60% of HCWs report exposure to potentially morally injurious events (PMIEs) [12,13]. More than half of HCWS exposed to PMIEs go on to develop downstream impairments in occupational, mental, and functional outcomes [14,15,16]. Emerging evidence shows that PMIE exposure in workplace environments are key drivers of turnover and burnout [17,18].

Addressing moral injury as part of HCW mental health support is a relatively new intervention focus [8]. Historically, there has been no shortage of efforts to develop individual mental health and well-being support interventions for HCWs [19]. Most existing mental and occupational health interventions for HCWs are limited in reach, acceptability, accessibility, and effectiveness, which restrict their ability to have positive impacts for HCWs [5,19,20,21]. Interventions with a one-to-one delivery format are insufficient due to a scarcity of trained mental health clinicians [22] juxtaposed by a high need [23] and the skepticism and stigma of seeking mental health support [24]. Additionally, individual interventions fail to address many of the system-level causes of dysfunction among HCWs and healthcare systems, such as increased workload, inadequate supplies, staffing shortages, changes in electronic health records, value discrepancies, lack of social support, institutional betrayal, the politicization of science, and the spread of misinformation, e.g., [16,25,26,27,28,29].

Despite the myriad of organizational and structural challenges facing HCWs, most are not interested in current individually focused workplace well-being interventions [1,30]. This mismatch may be driven by value discrepancies between healthcare workers and the organizations they serve [31], lack of work time to engage in the interventions, as well as feelings among HCWs that they must use personal time and money to solve problems that are derivative of workplace exposures to PMIEs and excessive workload. Relational coordination between HCWs is crucial to functioning healthcare systems, and we need organizational interventions (e.g., offered during work time, does not individualize structural problems, improves workplace culture/workload) to re-establish healthy and supportive social connections [9,32].

In the current study, we present an intervention pilot designed to help address moral injury via two overarching purposes. Purpose 1 was to target moral injury as a key problem among HCWs and healthcare systems. Purpose 2 was to target moral injury with a systems-level approach, to take a step beyond the limitations imposed by interventions that are conceptualized and delivered in an individual, one-to-one format.

This pilot was conducted using an Evidence-Based Quality Improvement (EBQI) process, carried out in the context of a medical intensive care unit (MICU) in a rural academic medical center. We intentionally partnered with a MICU for two reasons. First, intensive care environments by definition and mission provide care for patients with high acuity and complexity for healthcare decision-making, an environment that naturally cultivates a higher risk for PMIE exposures and moral injury. Second, within the specific project context, the MICU with which we collaborated was experiencing a higher turnover rate than the national average, and we partnered in a practical manner to help address this challenge. Finally, our specific application of the EBQI methodology allowed us to focus on two key deliverables that are reported in this study: (1) design and implement this intervention and (2) assess its feasibility, acceptability, and preliminary effectiveness.

## 2. Materials and Methods

### 2.1. Participants and Procedure

The quality improvement study context was a rural academic medical center, with 460 inpatient beds and a staff of more than 8000 thousand employees. Staffing and turnover challenges during the period that we launched this project included vacancy rates in overall nursing of 25%, with a 22% annual turnover rate. For comparison, the 2023 national average turnover rate for Registered Nurses (RNs) was reported by Nursing Service, Inc (NSI) to be 18.4%, with Critical Care RN turnover reported at higher rates of 19.4% [33].

An IRB acknowledgment was granted to administer and evaluate this intervention as a quality improvement (QI) initiative. MICU nurses (full-time, benefitted nurses, non-travelers) had the opportunity to voluntarily participate in this project one time per month for six months from July 2023 to December 2023 during regularly scheduled staff meetings. The voluntary intervention was offered virtually and synchronously for a total of 6 possible sessions. Nurses (including unit leadership) participated in the project during normal (paid) work hours by scheduling intervention sessions to take place during existing staff meetings and did not receive any additional incentives to participate. The intervention was led by a MICU nurse trained by the project team and supported by a mental health expert in a secondary co-facilitation role. The mental health experts varied, rotating between a chaplain, a psychologist, and an advanced practice psychiatric nurse practitioner. We used online self-report surveys administered via REDcap (Research Electronic Data Capture) [34,35] to collect effectiveness data at two-time points (entry in July of 2023, exit in December of 2023) and feasibility data at one time point (December of 2023). Staff meetings were offered three times per month, with nurses required to attend one staff meeting per month. During the project, the MICU employed 64 full-time equivalent (FTE) nursing staff. With approximately 50 FTEs being permanent staff, 76% of the MICU permanent staff attended at least one session of RECONN.

### 2.2. Intervention Design

Reflection and Connection (RECONN; the title of the intervention) was designed using an Evidence-Based Quality Improvement (EBQI) process [36]. Hempel and colleagues (2022) stated “EBQI aims to integrate scientific evidence and methods into the QI process while maintaining focus on team-based innovation and problem-solving within real-world settings”. EBQI informed our intervention development process in several ways (see Table 1). First, we used qualitative data from stakeholders to identify barriers and facilitators in the implementation context using components of the Consolidated Framework for Implementation Research (CFIR) [37]. To integrate current empirical knowledge with the specific needs of MICU nurses, we developed a semi-structured interview guide that we administered in individual interviews (*n* = 25) for a total of 25 current and former MICU nurses. Interview questions were designed to assess nurses’ experiences of moral injury, current sources of support, and barriers to seeking support for difficult workplace experiences. Former nurses were asked to answer these questions from their perspective of their experience in the MICU. Primary barriers and facilitators identified using CFIR domains are noted in Table 2.

Second, we identified and reviewed evidence-based interventions supported through clinical trials for moral injury conducted in military/veteran contexts, identifying the utility of particular content from Building Spiritual Strength [39,40]. One key change was the need for the intervention to address current and future PMIEs by staying embedded in organizational operations. Building Spiritual Strength was originally designed to support veterans who are processing past traumatic events and/or PMIEs, not continuing to face them day in and day out at work. Finally, we integrated skills and philosophy from an intervention entitled RE-WIRE (Re-Engaging Worthy Interpersonal Relationships), a third-wave Cognitive Behavioral Therapy approach designed to target social connection, polarization, and loneliness [41].

We note some of the highlights relative to the development of the RECONN interventions (full discussion of these domains will be reported in a future implementation science manuscript from the project). Teaming was noted as a unit strength through shared experience of nurses and the peer support they naturally offered to one another. Nurses also named nuanced desires for mental health or chaplain support related to the adapting construct. Benefits of chaplain support were noted because chaplains were known by nurses (had a routine presence on the unit) and shared the care provision alongside nurses with their patients. Structurally, healthcare chaplains have the dual role of both patient and staff care. Challenges to mental health support included lack of availability and trust in Employee Assistance Program (EAP) services as a barrier related to available resources.

Given these identified barriers, we designed RECONN as a co-facilitated intervention with a nurse, chaplain, and/or mental health professional. Second, RECONN was designed with the recognition that short staffing and limited time to access supportive resources were both barriers in the healthcare system at large and in the MICU specifically. RECONN sessions occurred “on the clock”, time when nurses are paid to engage in work-related activities and during an existing meeting. We collaborated with unit leadership, working together to identify an existing medium through which RECONN could be implemented (i.e., collaboratively identified as existing staff meetings) and adapted the staff meetings on frequency (increased to occur monthly), length (reduced to 1 h total meeting time), and structure (delegated the first 30 min for mandatory education/coordination and the last 30 min for voluntary participation in RECONN). Staff meetings were offered virtually “off shift” so that nurses were paid to join but were not needing to juggle patient care responsibilities.

#### Co-Facilitation and Nurse Support

Guided by interview findings about the limited mental health and staffing resources in the medical center at large, and the MICU specifically, we built RECONN implementation to titrate co-facilitator support from high support to minimal support for the nurse facilitators across the six-month implementation period. Specifically, during the first 3 months of RECONN, nurse facilitators met weekly in sessions with a mental health expert (and the project team at large) for consultation, support, and education; during the final 3 months of the implementation period consultation was performed ad-hoc, as needed. This titration was determined by protocol, nurse comfort with facilitating the intervention, and team evaluation of nurse facilitator fidelity. Additionally, involvement of the expert co-facilitator (chaplain, psychologist, nurse practitioner) began as a more active and modeling role (during months 1 and 2) and shifted to a more passive role (during months 3–6) used only for maintaining healthy structure and boundaries of the session. Our goals with structure and boundaries focused on reducing potential harms that might occur via invalidation or excessive advice-giving (e.g., from older more experienced nurses to younger less experienced nurses) and re-directing towards optimizing empathy, self-reflection, and communication of shared experience among the group participants.

The EBQI process enabled us to build RECONN from existing empirically supported treatments for moral injury and social connection while addressing the specific implementation barriers and facilitators identified in our interviews. The content of the RECONN intervention sessions (script and slides available upon request) included the following steps:

1. Facilitator identifies a person to share a challenging event (i.e., the sharer).
2. Facilitator prompts the sharer to share their challenging event.
3. Moment of silence and reflection for the larger group to consider shared experiences and personal feelings about the event.
4. Sharer asks the group the type of feedback desired (e.g., validation, deeper understanding, guidance).
5. Group members give feedback to the sharer.
6. Sharer discloses how they are feeling about the feedback and event.
7. Group is offered the opportunity to identify one or two individuals to reach out and check in with the sharer in the coming week.
8. Facilitator closes time together with a brief reflective reading or poem.

### 2.3. Measures

Primary outcome measures focused on feasibility and acceptability of the intervention, as well as trust and help-seeking behaviors. The response set for questions was “Completely Disagree, Disagree, Neither Agree or Disagree, Agree, Completely Agree”. Feasibility and acceptability questions included aspects of the intervention fit, applicability, and whether the intervention was doable. We utilized and adapted evidence-supported feasibility and acceptability measures [42,43]. Support questions included giving and receiving support from co-workers, ability to perform their job, and trust in co-workers.

Preliminary measures of effectiveness assessed coping, emotional recovery (an aspect of resilience), loneliness, and moral injury outcomes. Instrumental social support coping behaviors were measured with two items from the Brief COPE [44]; we used the Banik and colleagues [45] adaptations to these items for healthcare settings. Responses ranged from “I haven’t been doing this at all”, “I’ve been doing this a little bit”, “I’ve been doing this a medium amount”, to “I’ve been doing this a lot”. Emotional recovery was measured using an adaptation of Adair and colleagues’ four-item inventory [46]. Four items were included with responses ranging from “disagree strongly”, “disagree slightly”, “neutral”, “agree slightly”, to “agree strongly”. Loneliness was measured with the Short Scale for Measuring Loneliness in Large Surveys [47]. Three items were included with responses including “hardly ever”, “some of the time”, and “often”. Moral injury was measured with an adapted version of the Expressions of Moral Injury Scale [48]. Three questions assessed participants’ experiences with PMIEs. Responses included “disagree strongly”, “disagree slightly”, “neutral”, “agree slightly”, and “agree strongly”.

### 2.4. Analytic Approach

Descriptive statistics were compiled for feasibility and acceptability and trust and social support. Preliminary effectiveness was evaluated by computing effect sizes from paired *t*-tests changes in mean scores from pre- to post-intervention. All analyses were conducted in Stata Version 18.

## 3. Results

### 3.1. Demographic Characteristics and Participation

Participant characteristics are noted in Table 3. There were 16 nurses in our sample of pre/post measures with an average age of 37.4 years, 11.2 years of experience, and most participants were white (75%) and female (75%). Of note, one additional nurse completed the feasibility/acceptability measures who did not complete the initial measures, so that individual is not included in the sample characteristics. On average, 13 nurses attended each staff meeting over the six-month project, and we retained an average of 85% (11 nurses) who voluntarily stayed to attend RECONN sessions.

### 3.2. Feasibility and Acceptability of RECONN

Between 65–70% of nurses in the sample found RECONN to be fitting, applicable, and doable for nurses (Table 4). Though not accounted for in our survey instruments, there was initial hesitancy and skepticism about the intervention process that manifested after the first month. These concerns were raised individually with project leadership as well as during project team meetings; the project team in collaboration with the nurse facilitators and nursing unit leaders responded by adapting communication strategies in the introduction of RECONN sessions to address nurse participants’ negative experiences and concerns.

### 3.3. Trust and Social Support of RECONN

Most nurses were neutral (52.9%–58.8%) about the intervention impacting the trust and support they receive or provide and the intervention’s impact on their ability to perform their job (Table 5). More nurses tended to agree than disagree that this intervention improved their ability to perform their job (25% vs. 18.8%) as well as improved trust in co-workers (29.4% vs. 17.6%). More nurses reported disagreeing (23.5%) vs. agreeing (17.7%) with whether the intervention increased support they received from others.

### 3.4. Qualitative Feedback on RECONN

Nurses in the project provided qualitative feedback on what they liked, disliked, and would change about the intervention. Responses to what they liked about the intervention include the following: “The whole idea of a place to discuss morally distressing occurrences and receive support from others”; “It helped me to see that others are going through some of the same things”; and “Offers space to communicate difficulties seen at work with those who I feel have a good understanding”.

Responses about what they did not like about the intervention include the following: “Lack of participation from others. I also feel like some people do not follow through with follow-up support with those who share their thoughts with the group”; “It doesn’t change the environment we work in or the broken healthcare system. There is no fix while health care is a for profit industry run by insurance”; and “The lack of buy-in from people on the unit. Why would I want to continue to stay after staff meetings and discuss feelings when I constantly hear on the unit how no one wants to listen to others “whine about their feelings”?”.

Responses about what they would change included the following: “Make it available to the unit during distressing moments”; “I would just hope that others are more willing to participate in the sharing aspect and the follow up support”; and “Nothing I can think of. Maybe having it in person rather than virtually. The big issue for me was the lack of buy-in unfortunately and that’s nothing those running the project can change”.

### 3.5. Preliminary Effectiveness of RECONN

Though not statistically significant, preliminary efficacy data revealed promising effect size improvements in several outcomes (Table 6) of interest ranging from small (emotional recovery; −0.16) to moderate (coping; −0.44, loneliness; 0.45, moral injury; 0.46).

## 4. Discussion

MICU nurses had a high level of participation in the RECONN intervention, predominantly agreeing that the intervention was acceptable and feasible, and most participants gave a neutral response for trust and support constructs. The qualitative feedback named positive experiences, with opportunities for change in both organizational and unit-level culture. As an important note, the organization in which we implemented RECONN experienced a restructuring part way through the intervention implementation, which resulted in the layoff of several key stakeholders in the process (both in employee well-being and spiritual care). Despite a significant nurse engagement and design process, there was initial hesitation and resistance to the intervention implementation in the first month of the project. Organizational well-being interventions are difficult to implement, and nurse skepticism is likely a normative component of any change process in any health system.

HCWs are extremely resilient [4]. The underlying challenges for HCWs and healthcare systems will not be corrected by only building individual resilience-focused interventions. Such interventions are unwanted by the majority of HCWs, who are arguably already saturated with the health fluency and knowledge needed to optimize personal grit and mental health [1,30]. Overwhelmingly, HCWs favor interventions that can improve patient care and will likely benefit from efforts to align values across all echelons of healthcare organizations [30,31]. Our EBQI-informed intervention development project demonstrated that it is possible to develop an organizationally implemented intervention tailored to improve nurse well-being and with the potential to bridge value-discrepancy gaps within a hospital.

Deliberate intervention design was required to account for identified barriers and facilitators in an inpatient ICU context. Overall, most nurses involved in this project reported that the RECONN intervention was fitting and doable. Nurses reported mainly neutral results when asked about their feelings of trust and support related to the intervention. These neutral feelings have been due to existing mistrust in health system leadership that is not unique to any one healthcare organization, skepticism toward the longevity of a new well-being intervention, and/or nurse ambivalence.

We found small to moderate effects for RECONN improving coping, moral injury, loneliness, and emotional recovery in this quality improvement project. An organizational implementation of RECONN is underway, which will allow for a more robust evaluation of its effectiveness. Future versions of RECONN may need to incorporate more structured peer support elements between sessions to ensure appropriate follow-up support and intervention dosage.

Throughout the implementation of RECONN across the six-month period, iterative improvements were made to enhance engagement, effectiveness, and sustainability. The first improvement occurred after month one, wherein we encouraged “cameras on” for group members using a script that describes RECONN and “cameras on” as a way of producing more safety, engagement, support, and respect. The second improvement adapted the script to guide expressions of empathy and shared experience as opposed to problem-solving and advice-giving. The update addressed the observation of a preference for problem-solving and advice-giving over and above personal self-reflection and sharing of difficult experiences. This update was seen as especially important given the “unsolvability” of many difficult events related to being a nurse in the MICU (e.g., moral injury, workload, etc.) and the value of reflection on shared experience, empathy building, and peer support between nurses.

Though our intervention design process had notable strengths, it is limited in several ways. The project was conducted in one rural, academic medical center, which limits the generalizability of the RECONN intervention to other contexts. Implementation of the intervention at a unit level required significant staff and leadership investment (including changing the staff meeting schedule and format); units or health systems looking for “quick fixes” to unit culture and moral dilemmas likely will not have the mindset or capacity to successfully implement RECONN. Future RECONN evaluations should include other units, healthcare systems, and healthcare disciplines to ensure the generalizability of results beyond MICU nursing.

It is also critical to express the importance for healthcare leaders and individual medical centers to value, champion, and support rigorous research and interventions that can uncover and address pain points, moral injury, and trauma among modern-day healthcare workers. Progress in this area requires an organizational willingness towards vulnerability to uncover troubling and uncomfortable experiences of frontline HCWs. Such leadership and organizational buy-in was in place in the current study context within the academic medical center where we implemented RECONN, at unit and organizational levels. Without these supports, research and interventions in healthcare systems are difficult to carry out at best and perhaps impossible at worst.

Discussion about different constructs of healthcare worker distress is still an ongoing conversation in the field. We chose to work with moral injury as a construct for reasons outlined in the introduction. Moral distress is a similar (but different) construct that has been used historically in healthcare [49,50]. Lastly, organizational moral injury interventions remain relevant, but broader structural changes to improve health system staffing, lower administrative burden, increase autonomy, as well as bridge value discrepancies between senior leaders and direct care staff would likely bring greater improvements in HCW well-being [30,31].

## 5. Conclusions

RECONN was found to be feasible, acceptable, and doable by MICU nurses in a rural, academic medical center. Further research is indicated on the effectiveness and intervention adaptation for other healthcare professions and settings. Since the pilot project, a larger-scale project was funded that will enable future reporting on RECONN’s implementation and efficacy.

## Figures and Tables

**Table 1:** EBQI and RECONN Development.

EBQI Step	RECONN Project Step
Form a team of local partners and experts	Interdisciplinary team assembled from internal and external organizations
Prioritize determinants	The team identified turnover, moral injury, and unit culture as intervention priorities Individual interviews and focus groups sought additional insight from MICU nurses and stakeholders
Select EBI adaptations/implementation strategies	Existing literature was examined; interventions for adaption were chosen to meet identified concerns within constraints of the implementation environment
Specify and tailor EBI adaptations/strategies	RECONN was developed from existing interventions and tailored to the implementation context of the MICU
Refine EBI adaptations/strategies	Updates to RECONN were made throughout the quality improvement project

Table adapted from Swindle et al. 2023 [38].

**Table 2:** Barriers and facilitators of seeking support for moral injury using the Consolidated Framework for Implementation Research (CFIR).

CFIR Domain	CFIR Construct	Barriers and Facilitators	
		**Barrier (−)**	**Facilitator (+)**
**Intervention Characteristics**	Innovation Relative Advantage	(−)	
	Innovation Cost	(−)	
	Innovation Adaptability		(+)
**Characteristics of Individuals**	High-Level Leaders	(−)	
	Motivation		(+)
	Need		(+)
**Implementation Process**	Engaging	(−)	
	Teaming		(+)
	Adapting		(+)
**Inner Setting**	Work Infrastructure	(−)	
	Available Resources	(−)	
	Implementation Readiness	(−)	
	Tension for Change		(+)
**Outer Setting**	Local Conditions	(−)	

**Table 3:** Demographic characteristics of nurse participants (N = 16).

Demographic Characteristic	*n* (%) or Mean
Gender	
Female	12 (75.0)
Male	4 (25.0)
Race/Ethnicity	
White	12 (75.0)
Other racial groups	4 (25.0)
Average Age, years	37.4
Average Work Experience, years	11.2

**Table 4:** Feasibility and Acceptability (N = 17).

	*n*	%
**This intervention seems fitting for nurses**		
Agree	11	64.7
Neither Agree or Disagree	5	29.4
Disagree	1	5.9
**This intervention seems applicable for nurses**		
Completely Agree	1	6.3
Agree	10	62.5
Neither Agree or Disagree	5	31.3
**This intervention seems doable for nurses**		
Agree	12	70.6
Neither Agree or Disagree	5	29.4

**Table 5:** Trust and Social Support (N = 17).

	*n*	%
**Participation in this intervention has caused me to increase the support I provide to others:**		
Completely Agree	1	5.9
Agree	4	23.5
Neither Agree or Disagree	9	52.9
Disagree	3	17.7
**Participation in the intervention has increased the support I received from others:**		
Agree	3	17.7
Neither Agree or Disagree	10	58.8
Disagree	2	11.8
Completely Disagree	2	11.8
**Participation in this intervention has improved my ability to perform my job as a nurse:**		
Agree	4	25.0
Neither Agree or Disagree	9	56.2
Disagree	3	18.8
**Participation in this intervention has improved trust among my co-workers:**		
Agree	5	29.4
Neither Agree or Disagree	9	52.9
Disagree	2	11.8
Completely Disagree	1	5.9

**Table 6:** (**a**) Means and Standard Deviations Baseline and Post-Intervention (N = 16); (**b**) Preliminary Effect Sizes (N = 16).

(a)				
	**Mean ** **Baseline**	**SD** **Baseline**	**Mean** **Post**	**SD** **Post**
Instrumental Coping	4.5	1.26	5.06	1.29
Emotional Recovery	14.25	3.32	14.75	2.93
Loneliness	6.33	1.99	5.4	2.13
Moral Injury (participating/self)	7.75	2.49	6.56	2.68
**(b)**				
	**Effect Size**		**95% CI**	
Instrumental Coping	−0.44		−1.14, 0.27	
Emotional Recovery	−0.16		−0.85, 0.54	
Loneliness	0.45		−0.28, 1.17	
Moral Injury (participating/self)	0.46		−0.25, 1.16	

## Data Availability

This project did not create a publicly available dataset.
